# Capilliposide Isolated from *Lysimachia capillipes* Hemsl. Induces ROS Generation, Cell Cycle Arrest, and Apoptosis in Human Nonsmall Cell Lung Cancer Cell Lines

**DOI:** 10.1155/2014/497456

**Published:** 2014-01-08

**Authors:** Zheng-hua Fei, Kan Wu, Yun-liang Chen, Bing Wang, Shi-rong Zhang, Sheng-lin Ma

**Affiliations:** ^1^Department of Radiation Oncology and Chemotherapy, The First Affiliated Hospital of Wenzhou Medical University, No. 2 Fuxue Road, Wenzhou 325000, China; ^2^Zhejiang Chinese Medical University, No. 548 Binwen Road, Hangzhou 310053, China; ^3^Wenzhou Medical University, No. 58 Chashan Road, Wenzhou 325035, China; ^4^Department of Oncology, The Hangzhou First People's Hospital, No. 261 Huansha Road, Hangzhou 310006, China

## Abstract

Several data has reported that capilliposide, extracted from a traditional Chinese medicine, *Lysimachia capillipes* Hemsl. (LC) could exhibit inhibitory effect on cell proliferation in various cancers. The current study investigated the antitumor efficacy of *Capilliposide* and elucidated its potential molecular mechanism involved in vivo and vitro. Our results indicated that LC capilliposide inhibited proliferation of lung cancer cells in a dose-dependent manner. LC capilliposide induced cell cycle arrest at the S stage and enhanced apoptosis in NSCLC cells. Treatment with LC capilliposide increased the intracellular level of ROS, which activated the mitochondrial apoptotic pathway. Blockage of ROS by NAC highly reversed the effect of LC capilliposide on apoptosis. Xenograft tumor growth was significantly lower in the LC-treated group compared with the untreated control group (*P* < 0.05). The results also show that LC treatment does not produce any overt signs of acute toxicity in vivo. These findings demonstrate that LC capilliposide could exert an anti-tumor effect on NSCLC through mitochondrial-mediated apoptotic pathway and the activation of ROS is involved.

## 1. Introduction

Lung cancer has been the most common malignant tumor worldwide and the leading cause of human cancer-related deaths for several decades [1]. Nonsmall cell lung cancer (NSCLC) accounts for nearly 80% of lung cancer cases and approximately two thirds of these patients are diagnosed at an advanced stage. Chemotherapy or radiation therapy is largely ineffective and highly toxic with a low survival profile. Although the prognosis is improved by early diagnosis and treatment, tumor recurrence and progression still plague some patients [2]. Developing novel drugs and therapies with fewer side effects is of significance for prognosis of patients with NSCLC [3].

Reactive oxygen species (ROS) including superoxide anion, hydroxyl radicals, and hydrogen peroxide (H_2_O_2_) are produced by all aerobic cells, which had important role in variety of various biological processes during physiological and pathological conditions [4]. ROS are thought to play multiple roles in tumorigenesis, progression, and maintenance [5]. On the one hand, cancerous cells have shown a higher level of ROS compared with their noncancerous counterparts. Up-regulation of ROS is usually accompanied with oncogene activation which may contribute to cancer progression. On the other hand, an imbalance between production of ROS and antioxidant depletion results in irreversible oxidative stress. Anticancer drugs and ionizing radiation may be selectively toxic to cancer cells by increasing oxidant stress and enhancing the already stressed cells beyond their limit [6]. Intracellular ROS burst leads to cell cycle arrest and triggers apoptosis [7].

*Lysimachia capillipes* hemsl is a traditional medicinal plant that grows in southeastern China. The whole plant is used for treating coughs, menstrual, rheumatalgia disorder and carcinomas. Capilliposide had been extracted from *Lysimachia capillipes* by Tian et al. [8, 9]. Some experimental analysis have proven that LC capilliposide possess anti-cancer properties in different cancer cell lines both in vivo and in vitro, such as prostate and gastric cancer [10, 11]. LC capilliposide exhibited cytotoxicity against human breast cancer cells MCF7 with an IC50 value of 0.3 ug/mL [12]. Although capilliposidecan induce growth inhibition in cancer cells, the molecular mechanism underlying antitumor activity remained poorly understood. This study was, therefore, conducted to investigate the antiproliferative activity of LC capilliposide in nonsmall cell lung cancer (NSCLC) cell lines and its underlying mechanism.

## 2. Materials and Methods

### 2.1. Cell Cultures

The lung cancer cell lines A549, H1299, and H460 were obtained from Type Culture Collection of the Chinese Academy of Sciences (Shanghai, China). The cells were cultured in RPMI-1640 medium (Invitrogen, Carlsbad, CA, USA) supplemented with 10% fetal bovine serum (Gibco, Carlsbad, CA, USA). The cell lines were maintained in a humidified atmosphere containing 5% CO_2_ at 37°C. The culture medium was renewed every 2 to 3 days. Adherent cells were detached by incubation with trypsin. Throughout the experiment, the cells were used in logarithmic phase of growth.

### 2.2. Chemical Reagents and Antibodies

LC capilliposide was dissolved in double distilled water, presented by professor Tian from Zhejiang University (Hangzhou, China), TS101021. Dimethyl sulfoxide (DMSO), N-acetyl L-cysteine (NAC), cisplatin (DDP), 3-(4,5-dimethyl-2-thiazolyl)-2,5-diphnyl-2H-tetrazolium bromide (MTT), phenylmethylsulfonyl fluoride (PMSF), 5-(and 6)-carboxy-2′7′-dichlorodihydrofluorescein diacetate (DCFDA) and the fluorescent dyes Hoechst 33342, and propidium iodide (PI) were all purchased from Sigma-Aldrich (St. Louis, MO, USA). The monoclonal antibodies against p53 (number 2527), Bax (#5023P), cleaved caspase-3 (#9661s), cleaved caspase-9 (#9505p), cytochrome C (#11940S), GAPDH (#2118), and horseradish peroxidase (HPR)-conjugated goat antirabbit secondary antibody (#7074P2) were obtained from Cell Signaling Technology (Cell Signaling Technology, MA, USA). The monoclonal antibody against Bcl-2 (#sc-492) was obtained from Santa Cruz.

### 2.3. Cell Viability Assay

To evaluate the effect of LC capilliposide on A549, H1299, and H460 cell growth, cell viability was determined by MTT assay as described [13]. Cells were seeded in a 96-well microplate and treated with LC capilliposide at different concentrations (0–32 *μ*g/mL) for 24 h. After treatment, the MTT reagent was added (1 mg/mL) and cells were incubated for a further 4 h. Subsequently, 150 *μ*L DMSO was added to each well and the absorbance was measured in a microplate reader at the wave length of 570 nm (Thermo Electron Corp, Waltham, MA, USA). The percentage of cell viability was calculated as follows: cell viability (%) = A570 (sample)/A570 (control) × 100%. At least three replicates were performed for each treatment. The IC50 values were calculated using Graph Pad Prism 5.

### 2.4. Clonogenicity Assay

Clonogenicity assays were performed to determine the effects of LC capilliposide treatment on the colony-forming ability of H460 cells. Cells grew at low density, treated with LC capilliposide at different concentrations (0, 2, 4, and 6 *μ*g/mL) for 6 h. After cultured with fresh medium, cells were allowed to grow for 14 days to form colonies, which then were fixed and stainedwith 0.5% crystal violet (Sigma) in methanol for 30 min. The number of colonies (>50 cells) was scored using a microscopy.

### 2.5. Cell Cycle Analysis

H460 cells were seeded into 6-well culture plates. After LC capilliposide (0, 2, 4, and 6 *μ*g/mL) treatment for 24 h, the cells were collected and then fixed over night with 70% ethanol. After centrifugation, the cell pellets were incubated with 50 *μ*g/mL PI and 0.1% RNase in PBS for 30 min at room temperature in dark. The samples were measured using FACS flowcytometer (Becton Dicknson, USA).

### 2.6. Apoptosis Assay

Apoptotic cells by fluorescent staining were determined as previously described [14]. After LC capilliposide (0, 2, 4, and 6 *μ*g/mL) treatment for 24 h, the cells were stained with 5 *μ*L annexin V-fluoirescein isothiocyanate (FITC) and 10 *μ*L propidium iodide (PI) for 30 min at room temperature in dark. Stained cells were immediately measured using FACS Calibur flow cytometer and Cell Quest software.

### 2.7. Nuclear Double Staining with Hoechst 33342/PI

H460 cells were seeded into 6-well culture plates and treated with LC capilliposide (0, 2, 4, and 6 *μ*g/mL) for 24 h. After treatment, cells were harvested and washed with PBS. Hoechst 33342 (10 *μ*g/mL) was added, followed by PI (2.5 *μ*g/mL), and the cells were further incubated for 15 min at 37°C. Cells of blue and red fluorescence were examined under a fluorescence microscopy (Zeiss, LSM710, Germany) and 100 cells from five random microscope fields were counted.

### 2.8. Detection of Reactive Oxygen Species (ROS)

Intracellular ROS were measured using 2′,7′-dichlorodihydroluorescein diacetate (DCFH-DA). After incubation with LC capilliposide (0, 2, 4, and 6 *μ*g/mL) for 3 h, H460 cells (1 × 10^6^) were washed with PBS and labeled with 10 Um DCFDA for 30 min. Then, excess DCFH-DA was removed by washing the cells in serum-free 1640 RPMI medium. The fluorescence intensities were measured using an FACS flow cytometer.

### 2.9. Western Blot Analysis

After treatment with various concentrations of LC for 24 h, H460 cells were harvested, washed with PBS, and 0.1 mL of cold lysis buffer (150 mM NaCl, 50 mM of pH 7.4 Tris, 1 mM EDTA, 1% Triton X-100, 0.5% SDS, and 0.01% PMSF). The cell lysate was centrifuged at 4°C and 12000 ×g for 12 min, and the supernatant were collected. Protein concentrations were determined using the BCA protein assay (Beyotime Institute of Biotechnology, Jiangsu, China). Equal amounts of lysate (30 *μ*g) was subjected to 10 %SDS-PAGE at 80 mA and then transferred onto PVDF membranes. The membranes were blocked with a 5% skim milk solution for 1 h and incubated with respective primary antibodies overnight at 4°C. Then the membrane was incubated with a HRP-conjugated secondary antibody for 1 h at room temperature. The protein expression levels were determined by the enhanced chemiluminescence (ECL) system (ECL, Beyotime Institute of Biotechnology, Jiangsu, China).

### 2.10. In Vivo Studies

Female BALB/c nu/nu mice (16 weeks old, 18–20 g) were provided by Shanghai Experimental Animals Co. The animals were maintained at a specific pathogen-free grade animal facility with a regulated environment (22 ± 1°C, relative humidity 60 ± 5%) and a 12 h light and 12 h dark cycle (08:00–20:00, light). Then, H460 cells (1 × 10^7^) were subcutaneously inoculated into the right flank mice. Therapy was initiate 7 days after tumor inoculation when the mean tumor volume was 50 mm^3^. Tumor-bearing mice were divided into four groups (10 mice per group). Group of vehicle were infused with 100 uL physiological saline; group of L-LC were infused with 100 uL LC (40 mg/kg body weight) by oral administration; group of H-LC were infused with 100 uL LC (80 mg/kg body weight) by oral administration; group of DDP were administered with 100 uL cisplatin (1.2 mg/kg body weight) intraperitoneally. The groups of L-LC and H-LC were administered for 16 days, once a day. The DDP group was administered once every 2 days.

After 16 days of treatment, mice from each group were scarified and the weight of tumor mass was measured. The tumor weight of treatment group showed statistically significant differences compared with those of control group. No mice died during the period of treatment. Serum was separated and stored at −20°C for biochemistry analysis. In order to understand the acute side effect of LC treatment on liver and kidney function, the liver and kidneys were fixed in buffered formalin, embedded in paraffin, cut into 2 *μ*m sections, and stained with hematoxylin and eosin (H&E). Blood the biochemical parameters including alanine aminotransferase (ALT), aspartate aminotransferase (AST), albumin, gamma glutamyl transpeptidase (GGT), blood ureanitrogen (Bun), and creatinine (Cr) were measured by an automated biochemical analyzer (Hitachi 7600, Japan).

### 2.11. Statistical Analysis

Data are expressed as mean ± standard deviation. Statistical comparisons were performed using a one-way analysis of variance followed by the Fisher test. Significant differences between the groups were determined using an unpaired Student *t*-test.

## 3. Results 

### 3.1. LC Capilliposide Decreased Viability and Inhibited the Proliferation of NSCLC Cells

**Figure 1:**
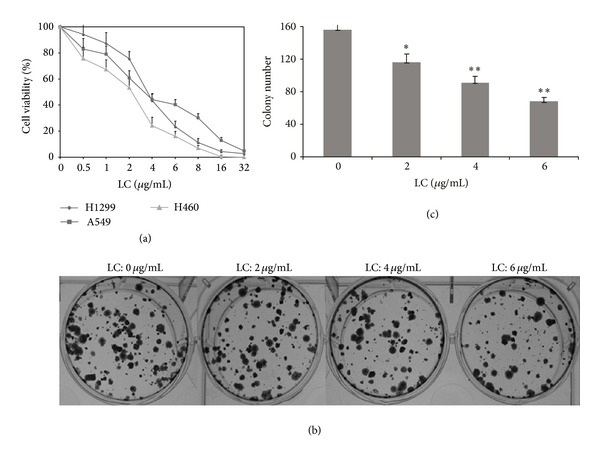
Effects of LC on cell viability and colony formation in of NSCLC cells. (a) Cell viability in LC-treated A549, H292, and H460 cells. The cells were treated with various concentrations (0–32 *μ*g/mL) of LC for 24 h. (b) Influence of LC on the number of colony-forming in H460 cells. Cells were treated with LC capilliposide (0, 2, 4, and 6 *μ*g/mL) for 6 h and allowed to grow for 14 days to form colonies. (c) Summary of colony-forming data in histogram form. All data are representative of at least three independent experiments. *P < 0.05 and **P < 0.01 indicate statistically significant differences versus control group.

In order to investigate the effect of LC capilliposide on cell viability of NSCLC cell lines, MTT assay was assayed using A549, H1299, and H460 cell lines.  Figure 1(a) indicated that cellular proliferation was inhibited by LC capilliposide for 24 hours in a dose-dependent manner. The IC50 values of LC capilliposide in A549, H1299, and H460 cells were 4.13 *μ*g/mL, 3.76 *μ*g/mL, and 2.85 *μ*g/mL, respectively. The IC50 values of LC capilliposide for 48 h in A549, H1299, and H460 cells were 3.54 *μ*g/mL, 2.61 *μ*g/mL, and 2.08 *μ*g/mL, respectively; the IC50 values of LC capilliposide for 72 h in A549, H1299, and H460 cells were 2.76 *μ*g/mL, 2.03 *μ*g/mL, and 1.58 *μ*g/mL, respectively. H460 cell lines seemed to be more sensitive to LC capilliposide. Thus, we selected H460 cell line as the model system to conduct mechanistic studies.

Clonogenic assays were performed to examine the long-term antiproliferative activity of LC capilliposide in H460 cells. As shown in Figures 1(a) and 1(c), the clone formation were 159 ± 13, 112 ± 10, 91 ± 10, and 68 ± 8 at the concentration of 0, 2, 4, and 6 *μ*g/mL LC capilliposide, respectively. In addition, the clonogenicity of H460 cell lines in the LC capilliposide groups was decreased in a concentration-dependent manner. LC treatment can significantly suppress the colony-forming activity compared the control group (*P* < 0.05). As clonogenic assays in vitro have been reported to correlate very well with in vivo assays of tumorigenicity in nude mice [15], we investigated the antitumor effects of capilliposide in vivo in the following test.

### 3.2. Capilliposide Causes Apoptosis and Cell Cycle Arrest

**Figure 2:**
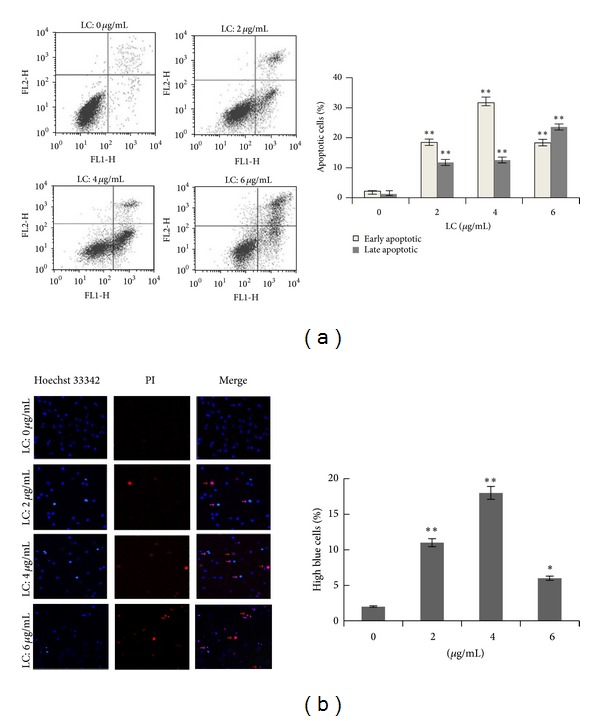
LC induces apoptosis in human NSCLC cells. (a) H460 cells treated with 2, 4 and 6 *μ*g/mL LC for 24 hours and apoptosis rates were analyzed by flow cytometry after annexin V/PI staining. (b) Apoptosis was assessed by Hoechst 33342/PI double staining as described in Section 2. High blue fluorescent indicates apoptotic cells (pink arrow), low blue indicates live cells (azury arrow), while red represents dead cells (red arrow). Apoptosis was expressed as a percentage of the total number of nuclei examined. All data are representative of at least three independent experiments. *P < 0.05 and **P < 0.01 indicate statistically significant differences versus control group.

To study the nature of LC-induced cell apoptosis, H460lung cancer cells were quantified with annexin V-FITC/PI double staining flow cytometry. As shown in Figures 2(a) and 2(b), LC capilliposide exposure at different concentrations (2, 4, and 6 *μ*g/mL) resulted in higher population of early apoptotic population (18.5 ± 1.8%, 31.7 ± 4.5% to 18.3 ± 2.6%, resp.) and late apoptotic population (11.8 ± 1.4%, 12.6 ± 2.1% to 23.6 ± 2.8%, resp.) compared to the control (*P* < 0.01). The data demonstrated that LC capilliposide induced a dose-dependent apoptosis.

To further understand the effect of LC on induced cell death, H460 cells were stained with Hoechst 33342/PI. Cells that were stained brightly by Hoechst 33342 were considered as early apoptotic cells. On the contrary, cells that were stained with both Hoechst 33342 and PI were considered to be at the late apoptosis. As depicted in Figure 2(b), there were higher percentages of bright blue cells (apoptosis) and red cells (necrosis). These data suggested that LC induced the apoptosis in H460 cells.

**Figure 3:**
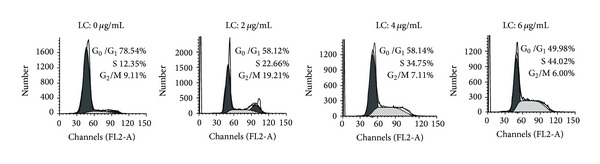
LC induces cell cycle arrest at S stage in H460 lung cancer cells. Cells were harvested and fixed in 70% alcohol and then stained with propidium iodide. Finally the stained cells were analyzed using a flow cytometer.

It has been reported that cell cycle arrest may induce apoptosis of cancer cells [16]. To evaluate the effect of capilliposide on the distribution of cell cycle, we performed DNA concentration in cell cycle analysis using flow cytometry. As shown in Figure 3, after 24 h treatment with capilliposide H460 cells were arrested in S-phase in a dose-dependent. Cells treated with 2, 4, or 6 *μ*g/mL LC capilliposide showed higher S population (22.66%, 34.75%, and 44.02%, resp.) compared with 12.35% in the control (*P* < 0.05). With the increase in the S-phase cell population, cell populations in the G_0_/G_1_ and G_2_/M phase decreased concomitantly.

### 3.3. LC Capilliposide Induced Apoptosis by ROS Generation in H460 Lung Cancer Cells

**Figure 4:**
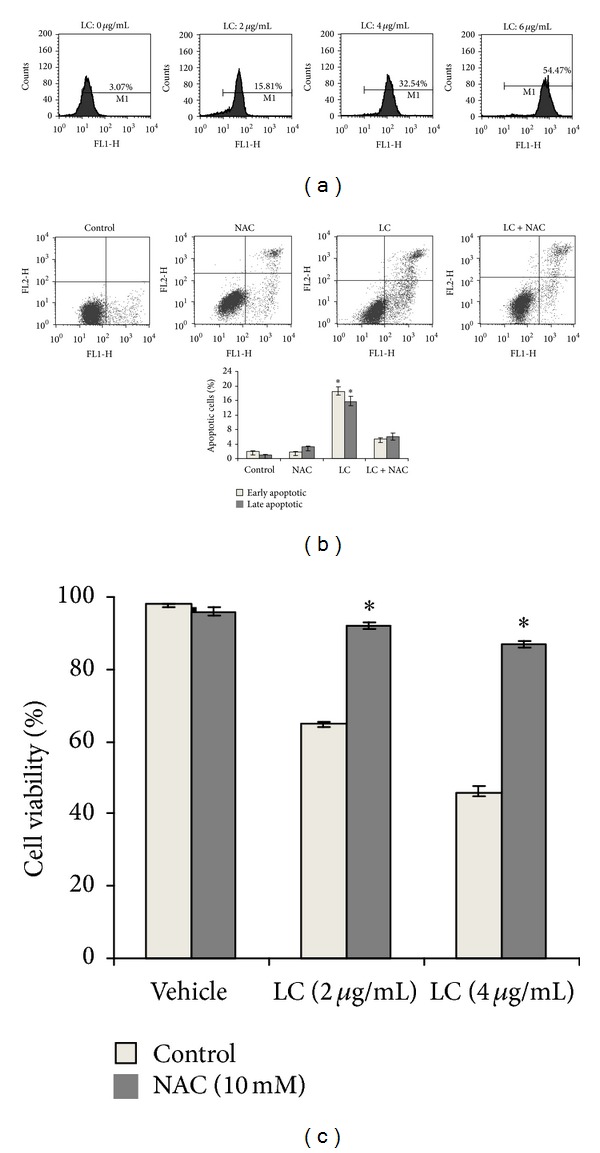
LC leads to ROS-mediated proliferative inhibition and apoptosis in H460 lung cancer cells. (a) ROS levels were determined 3 h later by flow cytometric analysis. The data demonstrated difference in the levels of intracellular ROS in control versus LC treated cells from a representative experiment. (b) The role of ROS in LC-induced apoptosis inhibition was assessed using ROS scavenger NAC. Cells were pretreatment with NAC (10 mM) for 1 h and then cotreated with 4 ug/mg LC for another 24 h. Impact of NAC on the apoptotic value was determined by annexin V-FITC/PI staining. (c) The role of ROS in LC-mediated proliferative inhibition was assessed using ROS scavenger NAC. The influence of NAC on LC-induced cytotoxicity was determined by MTT assay. All data are representative of at least three independent experiments. *P < 0.05 and **P < 0.01 indicate statistically significant differences versus control group.

Some reports have shown that the generation of ROS in intracellular could induce apoptosis by saponins extracted from different plants [17, 18]. Therefore, we hypothesized that LC capilliposide may cause H460 cells apoptosis via increased ROS production. To test this hypothesis, we investigated whether LC capilliposide treatment has associations with ROS burst in lung cancer cells. As shown in Figure 4(a), H460 cells in various concentration of LC capilliposide had a higher level of ROS-associated mean fluorescence intensity (MFI) compared with control. The level of ROS treated with 2, 4, and 6 *μ*g/mL LC correspondingly increased from 15.81 ± 2.63% to 32.54 ± 4.25% and 54.47 ± 6.21%. To further confirm that ROS was involved in LC capilliposide induced apoptotic pathway of H460 cells, NAC was used to scavenge the over production of ROS from both enzymatic and nonenzymatic mechanisms. Additionally, H460 cells were cultured with LC ± NAC (10 mM) for 24 h, then we analyzed cell viability by MTT and detected the apoptosis rate using Annexin V-FITC/PI double-labeled assay. As shown in Figures 4(b) and 4(c), our data demonstrated that treatment with NAC significantly inhibits the effect of LC capilliposide anti-proliferative and LC-induced apoptosis. Taken together, these results strongly support the hypothesis that LC-induced apoptosis via increased intracellular ROS oxidative stress in H460 cells.

#### 3.3.1. Effect of LC on Expression of Apoptotic-Related Proteins in Lung Cancer Cells

**Figure 5:**
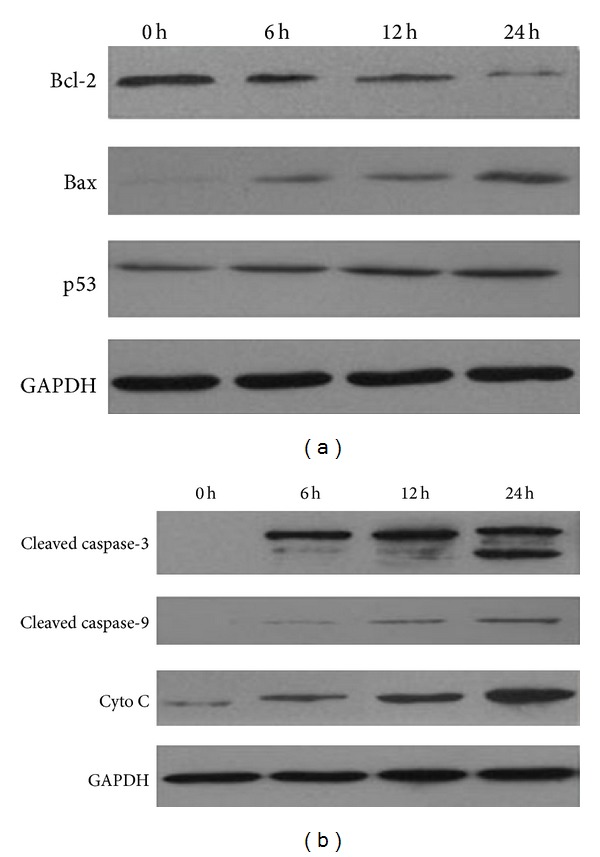
LC suppresses lung cancer cell growth via an apoptosis-independent mechanism. (a) Cells were treated with LC (4 *μ*g/mL) for 6 h, 12 h, and 24 h. Western blot assays were performed to determine the expression of Bcl-2, Bax, and p53 in H460 cells. GAPDH was used as a loading control. (b) Western blot assays were performed to determine the expression of cleaved caspase-3, cleaved caspase-9, and cytochrome C in H460 cells.

Our results in Figures 2 and 4 demonstrated that LC capilliposide induced cells apoptosis and intracellular ROS accumulation. ROS accumulation was described as an early event of mitochondrial apoptosis. So we hypothesized LC capilliposide induced apoptosis via a mitochondria dependent pathway. To verify this hypothesis, H460 cells were harvested after treatment with 4 *μ*g LC capilliposide for various time periods and total protein levels from each treatment were measured by Western blotting analysis. The Bcl-2 protein family plays a regulatory role in controlling the mitochondrial apoptotic pathway, including antiapoptotic (Bcl-2) and proapoptotic members (Bax) [19]. P53 as a tumor suppressor can regulate the expression of Bcl-2 and Bax protein to mediate mitochondrial apoptosis [20]. As shown in Figure 5(a), treatment with LC upregulated the expression of Bax and P53 whereas the expression of Bcl-2 was downregulated. Release of the cytochrome C from the mitochondria into the cytosol was a critical process for cells to undergo apoptosis. As shown in Figure 5(b), the level of protein cytochrome C, cleaved caspase-3 and cleaved caspase-9 was elevated after LC treatment in H460 cells. Western blotting analysis also showed that treatment with LC lead a time-dependent increase in the expression of cytochrome C, cleaved caspase-3 and 9 at 6 h, 12 h, and 24 h, suggesting a possible involvement of caspases activation in the apoptotic effect of LC in H460 cells in vitro.

### 3.4. LC Inhibited the Growth of Human H460 Xenografts

**Figure 6:**
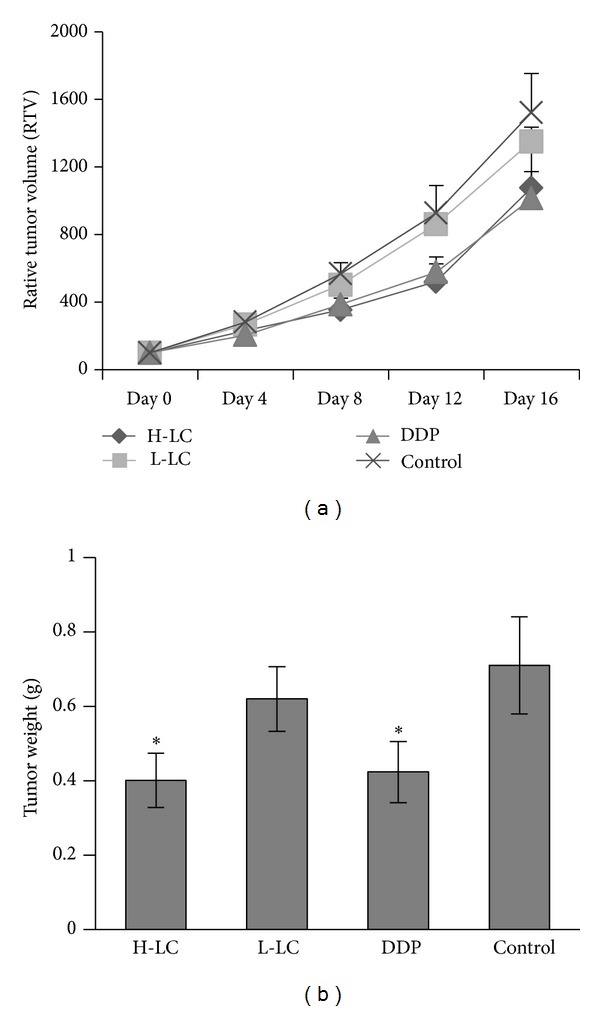
LC inhibited the growth of human H460 xenografts. After mice were injected with H460 cells (2 × 10^7^), they were divided into four groups. Control, L-LC (40 mg/kg) and H-LC (80 mg/kg) were administered orally, each day for 16 days. The DDP group (1.2 mg/kg) was injected once every 2 days. (b) Tumor weights. After 16 days of treatment, mice from each group were scarified and the weight of tumor mass was measured. Values were presented as mean ± SD, (*n* = 10). *P < 0.05 indicate statistically significant differences versus control group.

In order to access the therapeutic efficiencies of different concentrations of LC on H460 xenografts, body weights and the tumor sizes were measured by a caliper every 3 days in vivo. As shown in Figure 6, control group keep a rapid growth all the time due to the lack of anti-cancer drugs and the average relative tumor volumes (*V*/*V*_0_) was 15.20 ± 2.36; administration of LC (80 mg/kg) or cisplatin significantly decrease the size of tumor formation compared with control groups and their average relative tumor volumes (*V*/*V*_0_) were 10.75 ± 1.84 and 10.12 ± 2.41 compared with 15.20 ± 2.36 in the control (*P* < 0.05); LC (40 mg/kg) administration also resulted in growth suppression of H460 xenografts, but there is no significant difference compared to the control groups (*P* > 0.05). Consistent with the tumor volumes, tumor weights in Groups H-LC and DDP were significantly lighter than those in Group control (*P* < 0.05). These suggest that oral treatment of LC could significantly inhibit the development and progress of tumor formation in lung cancer model.

### 3.5. Acute Side Effects of LC

**Figure 7:**
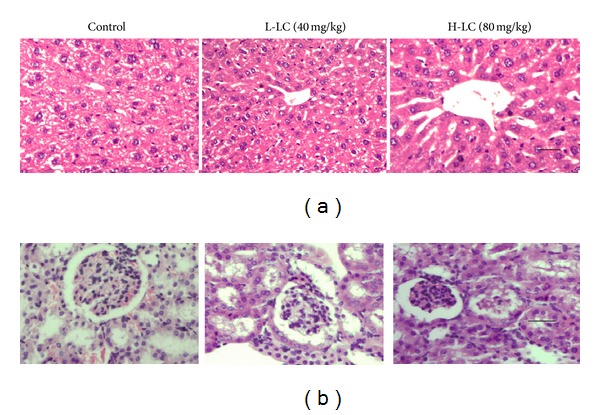
Effect of LC on mice liver and kidneys. The liver and kidneys from control and LC-treated mice were excised and processed for hematoxylin and eosin staining followed established procedures. (a) kidney section, scale bar = 100 *μ*m; (b) liver sections, scale bar = 100 *μ*m.

In order to understand acute side effects of LC, we investigated the changes in body weight, blood biochemistry, and histopathology of liver or kidney using BALB/c nu/nu mice. The four groups of mice were in good general state, body weights in the four groups had no obviously difference (*P* > 0.05, date not shown). The histopathological changes in liver and kidneys were assessed using hematoxylin and eosin staining. As shown in Figure 7, no obvious histopathological changes were observed in liver and kidneys structures of Groups L-LC and H-LC compared with control group.  Table 1 represents a comparison between the levels of ALT, AST, albumin, GGT, Bun, and Cr of control and treated groups of mice. The results show that the group DDP treated with cisplatin had a slight increase in serum Bun and Cr compared with control group, but the increase was not significantly different (*P* > 0.05). On the otherhand, groups treated with LC did not exhibit obvious hepatotoxicity or nephrotoxicity concerning the serum parameters compared with control group.

## 4. Discussion 

Lung cancer has long been the leading mortality in developed and developing countries. Due to limited efficacies of traditional radiation and chemotherapy, it is urgent to exploit new treatment strategies for lung cancer. An increasing amount of attention has been focused on the use of natural products isolated from Chinese medicinal herbs for lung cancer therapy [21, 22]. The major finding of the present study is that LC capilliposide, a natural compound extracted from *Lysimachia capillipes* Hemsl, effectively decreases human lung cancer cell viability via induction of apoptosis, which suppress the tumor proliferation both in vitro and in vivo. An ideal cancer chemotherapeutic agent must not only kill the cancer cells but must in addition exhibit a high degree of selective toxicity between cancer cells and normal cells [23]. The results also show that LC treatment does not produce any obvious signs of acute toxicity in vivo. It suggests that LC capilliposide may discriminate between normal and cancer cells.

Apoptosis, or programmed cell death, is an essential mechanism through which many types of chemotherapeutic agents inhibit tumor growth [24, 25]. As shown in Figure 2(a), cell population with annexin V positive and PI negative are considered as an early apoptotic population, whereas a cell population with both annexin V and PI positive is considered as a late apoptotic/necrotic population [26]. Our results firstly demonstrate that LC induces both early and late apoptosis in H460 cells in dose-dependent manner. In addition, cells undergoing apoptosis may lead to characteristic morphological changes, such as cell shrinkage, ruling, and chromatin condensation [27, 28]. So Hoechst 33342/PI double staining was used to identify the morphological changes in apoptotic nuclei. As shown in Figure 2(b), the nuclei of cells treated with LC was darkly stained, and thus fluoresced brightly, indicating the condensation of chromatin.

Mitochondria-initiated responses are thought to be the major pathway for apoptosis, and, therefore, targeting the mitochondria is a novel strategy for cancer therapy [29, 30]. Several genes are involved in the regulation of mitochondrial apoptosis, such as the Bcl-2 family and cytochrome C (cyto C). The Bcl-2 gene family, which is significantly involved in the regulation of cell apoptosis, both anti-apoptotic genes (Bcl-2, Bcl-XL) and proapoptotic members (Bax, Bak) [31]. The balance between the expression levels of pro- and anti-apoptotic proteins is critical for cell survival or cell death. Bcl-2 is an upstream effect or molecule in the apoptotic pathway and has been identified as a potent suppressor of apoptosis. As shown in Figure 4, LC treatment significantly downregulated Bcl-2 protein and upregulated levels of Bax protein in H460 cells, leading to an up regulation of the ratio between Bax and Bcl-2 [32]. This indicates the involvement of the Bcl-2 gene family. Furthermore, up-regulation of the ratio between Bax and Bcl-2 may induce the release of the cytochrome C from the mitochondria into the cytosol, which play a key role in the regulation of caspase-dependent cell death [33, 34]. Our results indicate that LC treatment significantly enhanced the release of the cytochrome C and increased the caspase-9 activity. Both intrinsic and extrinsic pathways converge on common factors including caspase-3. The activation of caspases-3 damages the cell structure and cause functional disorder by proteolysis, final induction of apoptosis. Our Western blot analysis also showed that caspase-9 and caspase-3 were all involved in LC-induced apoptosis in H460 cells. These results clearly indicate that LC induces apoptosis via mitochondrial pathways.

Apart from apoptosis, cell cycle arrest is another cause of growth inhibition. Many anti-cancer agents exhibit anti-proliferation by inhibiting cell cycle progression at a particular check point such as G_0_/G_1_, S, or G_2_/M [35, 36]. Deregulation of cell cycle has been linked with cancer initiation and progression. p53, a tumor suppressor protein, triggers cell cycle arrest to provide time for self-mediated apoptosis through transcriptional activation of cyclin-dependent kinase (CDK) inhibitor p21 [37, 38]. Data presented in Figure 5 showed a significant and progressive increase in the expression of p53 protein in LC-treated Cells. Interestingly, flow cytometry analysis also showed cell cycle was arrested at the S phase after treated with LC, suggesting that LC induce apoptosis of H460 cells via cell cycle arrest in S phase, which might be regulated by p53. Besides cell cycle arrest, p53 can induce the expression of several genes involved in apoptosis. For example, the Bcl-2 family has been shown to be a p53 target. An up-regulation of the ratio between Bax and Bcl-2 is involved in the regulation of p53-mediated cell death [39]. Our dates demonstrate that LC treatment increases the expression of Bax and p53 and decrease the expression of Bcl-2 by western blot assay. This suggests that p53 is involved in the apoptotic effect of LC.

ROS, which are the byproducts of normal cellular oxidative processes, have been suggested as regulating the process involved in the initiation of apoptotic signaling. Evidence is accumulating which indicates that many anticancer agents destroy tumor cells by raising the level of ROS above a toxic threshold [40–42]. High level of ROS can destroy the integrity of plasma membrane, affects dynamic of actin cytoskeleton and causes DNA damage, cumulatively known as oxidative stress. To investigate whether LC induce apoptosis is promoted through an increase in ROS production, we measured ROS levels using DCFH-DA staining and flow cytometric assays. The results showed that the apoptotic effect of LC on H460 cells was associated with increased ROS production. Moreover, to further confirm the finding that the apoptotic effect of LC was mediated by ROS, H460 cells were exposed to LC ± NAC and then analyzed for cell viability and apoptosis of H460cells [7]. As shown in Figure 4(b), treatment of H460 cells with NAC led to a significant reduction in LC-induced cell killing and apoptosis. Together, these results suggest that the accumulation of ROS is an important mechanism in the mitochondrial apoptosis pathway. However, the mechanism by which LC generates ROS needs to be further investigated in the future.

In summary, our data provide evidence for the first time that LC induces apoptosis in H460 NSCLC cells via ROS generation resulting p53 activation, increase Bax/Bcl-2 ratio, release of cytochrome C, and cleavage of caspases 9, 3. Finally, our data also showed that the growth of xenograft tumors was remarkably inhibited by oral administration of LC, indicating that the agent also has potential for clinical anticancer activity. Importantly, LC did not induce significant acute toxicity in mouse liver and kidneys. LC therefore has the potential to be a potent agent for non-small lung cancer treatment.

## Figures and Tables

**Table 1:** Effect of LC on blood biochemistry of control and treated mice.

Group	AST (U/L)	ALT (U/L)	Albumin (U/L)	GGT (U/L)	Cr (umol/L)	BUN (mmol/L)
Control	95.4 ± 7.6	38.3 ± 4.2	27.3 ± 3.2	2.1 ± 0.4	22.3 ± 2.5	9.4 ± 1.1
LC (40 mg/kg)	87.6 ± 6.5	36.6 ± 5.1	30.4 ± 3.5	2.6 ± 0.6	22.6 ± 3.8	8.6 ± 1.4
LC (80 mg/kg)	104.8 ± 8.4	43.7 ± 6.1	25.8 ± 2.7	2.9 ± 0.7	24.1 ± 3.5	10.8 ± 1.5
DDP	106.2 ± 7.9	46.5 ± 5.8	24.6 ± 2.8	2.8 ± 0.3	28.4 ± 4.2	12.3 ± 2.2
